# Lesiones del seno maxilar y su asociación con lesiones apicales observadas por tomografía computarizada de haz cónico. Un estudio transversal retrospectivo

**DOI:** 10.21142/2523-2754-1101-2023-139

**Published:** 2023-03-26

**Authors:** Manuela Alejandra Rodríguez López

**Affiliations:** 1 Facultad de Odontología, Universidad de Carabobo. Carabobo, Venezuela. manuela.rodriguezlopez@gmail.com Universidad de Carabobo Facultad de Odontología Universidad de Carabobo Carabobo Venezuela manuela.rodriguezlopez@gmail.com

**Keywords:** engrosamiento de la mucosa sinusal, seno maxilar, enfermedades periapicales, tomografía computarizada de haz cónico, sinus mucosal thickening, maxillary sinus, periapical diseases, cone beam computed tomography

## Abstract

**Introducción:**

Por medio de la tomografía computarizada de haz cónico pueden observarse alteraciones en los senos maxilares, como opacidades, ocupación de espacio y engrosamiento de la mucosa. Existen factores que contribuyen a dicho engrosamiento, entre los que destacan los factores dentales, la periodontitis, la patología apical y los tratamientos endodónticos.

**Objetivo:**

Evaluar la asociación entre cambios observados en los senos maxilares y lesiones apicales mediante tomografía computarizada de haz cónico.

**Materiales y métodos:**

Fue un estudio descriptivo con un diseño retrospectivo y transversal, correlacional, de campo, no experimental. La muestra estuvo conformada por 115 volúmenes tomográficos obtenidos por medio de un equipo Planmeca ProMax 3D Classic (Planmeca, Helsinki, Finlandia). Se evaluó la presencia/ausencia de tratamiento endodóntico en los dientes posteriores presentes, presencia/ausencia de lesión periapical asociada con estos dientes, tamaño de la lesión periapical, presencia/ausencia de alteración en el seno maxilar y su espesor.

**Resultados:**

Se observaron lesiones apicales que promediaron un tamaño de 3,32 ± 1,82 mm, y casi la mitad (44,35%) presentó entre 2 y 4 mm de tamaño. La principal alteración del seno maxilar que se observó fue el engrosamiento de la mucosa (58,26%). El espesor promedio del engrosamiento de la mucosa sinusal fue de 3,51 ± 1,78 mm, con un 72,17% de los casos con engrosamientos mayores a 2 mm.

**Conclusión:**

Hubo asociación entre los cambios observados en los senos maxilares y lesiones apicales. Mientras más grande y cercana estaba la lesión respecto del seno, mayor era el engrosamiento de la mucosa sinusal.

## Abstract (en)

**Introduction:**

Through cone beam computed tomography, alterations in the maxillary sinuses, such as opacities, space occupation and thickening of the mucosa, can be observed. Some factors contribute to this thickening, standing out among dental factors, periodontitis, apical pathology and endodontic treatments.

**Objective:**

To evaluate the association between changes observed in the maxillary sinuses and apical lesions using cone beam computed tomography.

**Materials and methods:**

It was a descriptive study with a retrospective and cross-sectional, correlational, field, non-experimental design. The sample consisted of 115 tomographic volumes obtained using Planmeca ProMax 3D Classic equipment (Planmeca, Helsinki, Finland). The presence/absence of endodontic treatment in the present posterior teeth, presence/absence of periapical lesion associated with these teeth, the size of the periapical lesion, presence/absence of alteration in the maxillary sinus and its thickness were evaluated.

**Results:**

Apical lesions were observed that averaged a size of 3.32 ± 1.82 mm, and almost half (44.35%) presented between 2 and 4 mm in size. The main alteration of the maxillary sinus that was observed was the thickening of the mucosa (58.26%). The average thickness of the thickening of the sinus mucosa was 3.51 ± 1.78 mm, with 72.17% of the cases with thickening greater than 2 mm.

**Conclusion:**

There was an association between the changes observed in the maxillary sinuses and apical lesions. The larger and closer the lesion was to the sinus, the greater the thickening of the sinus mucosa.

## INTRODUCCIÓN

Los senos maxilares son las cavidades sinusales más grandes y los primeros en formarse. Con un volumen de 60-80 mm^3^, ocupan bilateralmente el cuerpo del maxilar superior y crean una limitación de cuatro paredes; su pared medial es la más compleja ^1^^-^^4^. Su función es calentar el aire que entra mediante las fosas nasales, la fonación y la aireación. Presentan una forma de pirámide irregular con la base dirigida hacia la fosa nasal y el vértice orientado al hueso malar ^5^. Sin embargo, su morfología varía entre individuos e, incluso, en una misma persona, por lo que pueden ser completamente diferentes. Su drenaje se realiza a través del ostium nasal, con lo que obtiene ventilación a través de esta vía y mantiene su balance biológico; por ello, su obstrucción causaría la disminución de la ventilación y el drenaje ^6^^-^^8^.

Los senos maxilares son los de mayor importancia entre los senos paranasales, por su íntima relación con el proceso dentoalveolar. Radiológicamente, pueden observarse en los senos maxilares diferentes alteraciones que se manifiestan como opacidades parciales o totales, ocupación de espacio y engrosamiento de la mucosa ^3^^,^^9^^-^^11^.

Cabe mencionar que la membrana de Schneiderian reviste la cavidad nasal y los senos maxilares; asimismo, está involucrada en factores alterados en el momento de la evaluación radiológica y puede facilitar el diagnóstico, el pronóstico y el plan de tratamiento ^12^. Diversos estudios han analizado los factores que potencialmente contribuyen al engrosamiento de la mucosa del seno. Entre los factores dentales, la periodontitis, la patología apical y los tratamientos endodónticos fueron las causas predominantes de dicho engrosamiento que, con mayor frecuencia, producía una ocupación entre 2 y 10 mm ^13^^,^^14^. 

La íntima relación de las piezas dentales superiores con los senos maxilares implica que se debe tener cuidado al realizar procedimientos endodónticos y quirúrgicos. Un descuido puede ocasionar alteraciones de manera directa, así como rinosinusitis, rinosinusitis crónica, quiste de retención, mucocele, entre otros ^15^^-^^18^. 

Las lesiones apicales son de tipo inflamatorio y acompañan procesos crónicos que suelen ser generados por la presencia de microorganismos invasores en el tejido periapical del conducto radicular. Suelen afectar a pacientes de la tercera edad o pacientes tratados endodónticamente. No confrontar a tiempo estas enfermedades pulpares puede traer consecuencias como la necrosis pulpar y la diseminación del proceso infeccioso a los tejidos periapicales a través del foramen apical. Según la asociación Americana de Endodoncia, a nivel periapical, se producen diversos cambios desde la parte histopatológica que se caracterizan como hiperemia, edema del ligamento periodontal, extravasación de neutrófilos, congestión vascular, los cuales son dirigidos mediante quimiotaxis hacia el área ^19^^-^^21^. 

De acuerdo con lo expuesto, el propósito de esta investigación fue evaluar la asociación de las lesiones apicales y los cambios observables en los senos maxilares mediante una tomografía computarizada de haz cónico. 

## MATERIALES Y MÉTODOS

Esta investigación descriptiva, retrospectiva y transversal fue aprobada por el comité de ética de la Universidad Científica del Sur, Lima, Perú con el número de registro 751-2020-POS70. Los pacientes firmaron un consentimiento informado con el cual autorizaron el uso de las imágenes exclusivamente con fines científicos. Asimismo, se garantizó el anonimato de los pacientes, de acuerdo con los lineamientos de la Declaración de Helsinki. La población estuvo conformada por imágenes de tomografía computarizada de haz cónico pertenecientes a pacientes de ambos sexos, mayores de 18 años, referidos al Centro de Imagenología de Especialidades Maxilofaciales (CIDEM), en Valencia, Venezuela, durante el periodo de enero de 2017 a diciembre de 2020. Se seleccionó una muestra a conveniencia comprendida por 115 volúmenes tomográficos, obtenidos por indicación clínica, de manera que ningún paciente fue expuesto a radiación ionizante con fines exclusivos para este trabajo. 

Se establecieron como criterios de inclusión los siguientes: tomografías que permitieran observar los senos maxilares, tomografías de pacientes con presencia de premolares o molares superiores en algún cuadrante, y ausencia de artefactos de imagen que interfieran con la evaluación. Los criterios de exclusión fueron tomografías de pacientes edéntulos, parcialmente edéntulos con ausencia bilateral de premolares y molares, presencia de artefactos y estudios de campo reducidos que no permitieran la evaluación de los senos maxilares.

Las imágenes tomográficas fueron obtenidas por medio de un equipo Planmeca ProMax 3D Classic (Planmeca; Helsinki, Finlandia) de sensor digital, con un FOV (*Field of View*) de 8 x 8, utilizando en medial de 90 kV, 10 mA, y tiempo de exposición de 18,4 segundos. Para la obtención de las imágenes se utilizó el siguiente protocolo: paciente en máxima intercuspidación dental, labios en reposo, mirada al horizonte y plano de Frankfort paralelo al piso. El análisis de las imágenes se realizó mediante el *software* Romexis versión 6.0 (Planmeca; Helsinki, Finlandia) y empleando un monitor. Los volúmenes tomográficos se analizaron en un monitor Apple modelo iMac de 21.5 pulgadas en un espacio de luz moderada. La evaluadora ajustó el contraste y brillo de la imagen para una óptima visualización cuando fue necesario. 

La investigadora fue entrenada por un radiólogo bucal y maxilofacial con experiencia en lo relacionado con la determinación de la presencia de lesiones apicales o lesiones existentes en el seno maxilar, así como en la medición de variables cuantitativas. Se registraron los siguientes datos: número y tipo de dientes posteriores presentes, presencia/ausencia de tratamiento endodóntico en los dientes posteriores presentes, presencia/ausencia de lesión periapical asociada con estos dientes, tamaño de la lesión periapical, distancia de la lesión periapical a la cortical del seno maxilar, estado de la cortical inferior del seno maxilar, presencia/ausencia de alteración en el seno maxilar, espesor del engrosamiento mucoso del seno maxilar en la zona del diente con lesión periapical.

Para el análisis de los datos se calcularon los estadísticos descriptivos media aritmética (x̄) y desviación estándar (DE) para las variables cuantitativas antropométricas, dentales y maxilares y se construyeron los intervalos al 95% de confianza para la media poblacional [IC95%], tanto en forma general como para las variables cuantitativas relativas a las lesiones de los senos maxilares clasificadas por las variables categóricas sexo, grupo etario, seno maxilar, tratamiento endodóntico, unidad dentaria afectada y tamaño de la lesión apical categorizado. La comparación entre grupos se realizó con la prueba t de Student para dos grupos independientes respecto de las variables cuantitativas relativas a las lesiones de los senos maxilares clasificadas por sexo, seno maxilar y presencia de tratamiento endodóntico; y con el análisis de varianza (ANOVA) y la prueba de comparación múltiples de Tukey para las mencionadas variables clasificadas por grupo etario, unidad dentaria afectada y tamaño de la lesión apical categorizado.

Se calcularon las distribuciones de frecuencia absolutas y relativas (%) para las variables cualitativas categorizadas antropométricas, dentales y maxilares, y se construyeron los intervalos de confianza al 95% para las frecuencias relativas. Se calculó el coeficiente de correlación de Pearson (r) para las variables cuantitativas relativas a las medias antropométricas y dentales con las variables cuantitativas relativas a las lesiones en los senos maxilares, y para los pares de variables que resultaron estadísticamente significativos se construyeron los gráficos de dispersión, a fin de caracterizar las correlaciones detectadas.

Los datos se procesaron con los programas estadísticos EPI INFO 7.2.4 (frecuencias absolutas y relativas e intervalos al 95% para las frecuencias relativas), Minitab 20.4 (estadísticos descriptivos, intervalos al 95% confianza para la media poblacional, prueba t de Student, ANOVA y prueba de comparaciones de media de Tukey, coeficiente de correlación de Pearson y gráficos de dispersión) y SPSS 26.0 (prueba de independencia de χ2).

## RESULTADOS

La muestra estuvo constituida por los resultados imagenológicos de 115 pacientes, 80 (69,57%) de sexo femenino y 35 (30,43%) de sexo masculino, con edades comprendidas entre 21 y 78 años, x̅ = 46,91 ± 15,53 años. Del total, 45 (39,13%) eran menores de 40 años, 40 (34,78%) tenían entre 40 y 59 años, y el resto (30; 26,09%) tenían 60 o más años. Las muestras contemplaron 77 (66,96%) resultados imagenológicos del seno maxilar derecho y 38 (33,04%) del seno maxilar izquierdo.

Con relación a las unidades dentarias analizadas, se encontró que los pacientes presentaban un promedio de x̅ = 5,57 ± 1,83 unidades dentales posteriores superiores, y de estas estaban afectadas (con lesiones apicales) un promedio de x̅ = 1,30 ± 0,53. Las unidades dentales analizadas fueron 22 (19,13%) segundos premolares [SPM], 79 (68,70%) primeros molares [PM] y 14 (12,17%) segundos molares [SM]. De estas unidades dentales, 45 (39,13%) presentaron tratamiento endodóntico. Las lesiones apicales observadas presentaron un tamaño promedio de x̅ = 3,32 ± 1,82 mm, y de estas, 35 (30,43%) tenían un tamaño de más de 1 hasta 2 mm, 51 (44,35%) más de 2 hasta 4 mm, y el resto, (29; 25,22%) más de 4 hasta 8 mm. 

Respecto de los resultados atinentes a las lesiones observadas en los senos maxilares, se tuvo que la distancia promedio de la lesión apical al seno maxilar fue de x̅ = 1,74 ± 0,87 mm, para 33 (28,70%) pacientes la distancia fue 0 mm o las lesiones se mostraron yuxtapuestas, para 37 (32,17%) pacientes la distancia fue mayor a 0 y menor a 2 mm, mientras que para el resto (45; 39,13%) fueron mayores a 2 mm. La alteración del seno maxilar predominante fue el engrosamiento de la mucosa (67; 58,26%); las alteraciones restantes presentaron frecuencias menores a 15% y 13 (10,43%) pacientes no mostraron alteración. Los estatus corticales más frecuentes fueron cortical adelgazada (45; 39,13%) y borrada (30; 26,09%); además, 21 (18,26%) pacientes no presentaron alteración. El espesor promedio del engrosamiento del seno maxilar fue x̅ = 3,51 ± 1,78 mm, observándose 9 (7,83%) pacientes sin engrosamiento, 23 (20%) con engrosamiento mayor a 0 y menor a 2 mm, y el resto (83; 72,17%) con engrosamientos mayores a 2 mm (Tabla 1).

**Tabla 1:** Características antropométricas, dentales y maxilares de los pacientes

Variable	Categoría	Frecuencia absoluta	Frecuencia relativa (%)	IC95%
Sexo	Femenino	80	69,57	60,29-77,80
	Masculino	35	30,43	22,20-39,71
Edad* (años)	46,91 ± 15,53		44,04 - 49,78	
Grupo etario (años)	20 a 39	45	39,13	30,16-48,67
	40 a 59	40	34,78	26,14-44,23
	60 o más	30	26,09	18,34-35,10
Seno maxilar	Derecho	77	66,96	57,57-75,44
	Izquierdo	38	33,04	24,56-42,43
N.^o^ de unidades dentarias posteriores*	5,57 ± 1,83		5,24-5,91	
N.^o^ de unidades dentarias posteriores afectadas*	1,30 ± 0,53		1,21-1,40	
Unidad dentaria afectada	SPM derecho	15	13,04	7,49-20,60
	SPM izquierdo	7	6,09	2,48-12,14
	PM derecho	51	44,35	35,09-53,91
	PM izquierdo	28	24,35	16,83-33,23
	SM derecho	11	9,57	4,87-16,47
	SM izquierdo	3	2,61	0,54-7,43
Unidad dentaria afectada	SPM	22	19,13	12,39-27,52
	PM	79	68,70	59,38-77,02
	SM	14	12,17	6,82-19,58
Tratamiento endodóntico	Sí	45	39,13	30,16-48,67
	No	70	60,87	51,33-69,84
Tamaño de la lesión cortical* (mm)	3,32 ± 1,82		2,98 - 3,66	
Categoría del tamaño de la lesión cortical (mm)	1 a 2	35	30,43	22,20-39,71
	2 a 4	51	44,35	35,09-53,91
	4 a 8	29	25,22	17,58-34,17
Distancia de la lesión al seno maxilar* (mm)	1,74 ± 0,87		1,58-1,90	
Categoría de la distancia de la lesión cortical al seno maxilar (mm)	0 o yuxtapuesta	33	28,70	20,65-37,88
	0 a 2	37	32,17	23,77-41,53
	2 o más	45	39,13	30,16-48,67
Tipo de alteración del seno maxilar	Sin Alteración	13	10,43	6,16-18,55
	Engrosamiento de la mucosa	67	58,26	48,70-67,39
	Poliposis	17	14,78	8,85-22,61
	Quiste mucoso	14	12,17	6,82-19,58
	Opacificación no específica	4	3,48	0,96-8,67
Estatus cortical inferior del seno maxilar	Sin alteración	21	18,26	11,67-26,55
	Adelgazada	45	39,13	30,16-48,67
	Borrada	30	26,09	18,34-35,10
	Desplazada	6	5,22	1,94-11,01
	Discontinua	13	11,30	6,16-18,55
Espesor del engrosamiento mucoso del seno maxilar* (mm)	3,51 ± 1,78		3,18-3,84	
Categoría del engrosamiento mucoso del seno maxilar (mm)	Sin alteración	9	7,83	3,64-14,34
	0 a 2	23	20,00	13,12-28,48
	2 a 4	40	34,78	26,14-44,23
	4 a 10	43	37,39	28,55-46,90

* Para las variables cuantitativas se muestran la media, la desviación estándar y el intervalo al 95% para la media poblacional.

**Figura 1:**
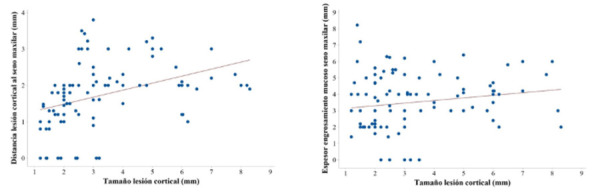
Gráficos de dispersión. De arriba hacia abajo: A) Tamaño de la lesión cortical (mm) y distancia de la lesión a cortical inferior del seno maxilar (mm); B) Tamaño de la lesión cortical (mm) y espesor del engrosamiento mucoso del seno maxilar (mm).

El coeficiente de correlación de Pearson indicó que hubo correlación estadísticamente significativa al 5% para el tamaño de la lesión cortical con la distancia de la lesión a cortical inferior del seno maxilar (r = 0,405; p<0,001) y al 10% con el espesor del engrosamiento mucoso del seno maxilar (p = 0,163; p = 0,083). En ambos pares de variables, el coeficiente de correlación presentó signo positivo, lo cual indica que la correlación detectada es directamente proporcional, es decir, a mayor tamaño de la lesión apical se esperan mayores distancias a la cortical inferior del seno maxilar y mayores engrosamientos del seno maxilar (Tabla 2). Esta asociación creciente o directamente proporcional puede apreciarse en los gráficos de dispersión mostrados en la Figura 1, en donde se evidencia la tendencia creciente para ambos pares de variables. No se detectaron correlaciones estadísticamente significativas para el resto de las variables cuantitativas consideradas.

**Tabla 2:** Correlaciones de Pearson entre las variables cualitativas antropométricas, dentales y maxilares

Variable	Distancia de la lesión a cortical inferior del seno maxilar (mm)		Espesor del engrosamiento mucoso del seno maxilar (mm)	
	Coeficiente de correlación	p	Coeficiente de correlación	p
Edad (años)	-0,099	0,294	-0,074	0,432
Número de piezas dentales posteriores	0,043	0,648	0,070	0,454
Número de piezas dentales posteriores afectadas	-0,059	0,533	0,024	0,803
Tamaño de lesión cortical (mm)	0,405	<0,001*	0,163	0,083

* significativo

El ANOVA indicó que solo hubo diferencias estadísticamente significativas al 5% para la distancia de la lesión cortical al seno maxilar, al ser clasificada por el tamaño de la lesión (p < 0,001). Asimismo, la prueba de comparación de medias de Tukey indicó que las lesiones con tamaños mayores a 4 mm presentaron la mayor distancia a la cortical del seno maxilar (grupo A de Tukey), mientras que las otras dos categorías presentaron un comportamiento homogéneo (grupo B de Tukey), es decir, tamaños promedios de lesión estadísticamente homogéneos entre sí y menores al grupo A. No se detectaron diferencias estadísticamente significativas para el resto de las variables (Tabla 3).

**Tabla 3:** Estadísticos descriptivos para la distancia de la lesión cortical al seno maxilar (mm) clasificado por las variables antropométricas y dentales

Variable	Categoría	n	Media	DE	IC95%	P
Sexo	Femenino	80	1,71	0,89	1,52-1,90	0,559
	Masculino	35	1,81	0,80	1,52-2,10	
Grupo etario (años)	20 a 39	45	1,89	0,90	1,64-2,14	0,106
	40 a 59	40	1,51	0,91	1,24-1,78	
	60 o más	30	1,81	0,69	1,51-2,12	
Seno maxilar	Derecho	77	1,76	0,92	1,57-1,96	0,662
	Izquierdo	38	1,69	0,75	1,41-1,97	
Pieza dental	SPM	22	1,62	1,04	1,25-1,99	0,773
	PM	79	1,76	0,86	1,57-1,96	
	SM	14	1,78	0,62	1,31-2,24	
Tratamiento endodóntico	Sí	45	1,83	0,96	1,58-2,09	0,370
	No	70	1,68	0,80	1,47-1,88	
Tamaño de la lesión apical (mm)	1-2	35	1,14B	0,63	0,89-1,39	<0,001*
	2-4	51	1,85A	0,90	1,64-2,06	
	4-8	29	2,27A	0,61	1,99-2,54	

*significativo

Letras diferentes significa diferencia significativa

La prueba de independencia de chi cuadrado indicó que hay asociación estadísticamente significativa entre las categorías de la distancia de la lesión apical a la cortical del seno maxilar con el tamaño de la lesión (p < 0,001). No se encontró asociación estadísticamente significativa con el resto de las variables (Tabla 4). Esta misma prueba indicó que hay asociación estadísticamente significativa entre el tipo de alteración del seno maxilar con el tamaño de la lesión (p < 0,001). No se encontró asociación estadísticamente significativa con el resto de las variables (Tabla 5). Se encontró asociación estadísticamente significativa al 5% entre el estatus de la cortical inferior del seno maxilar con el sexo de los pacientes (p = 0,012) y el tamaño de la lesión (p < 0,001), y estadísticamente significativa al 10% con la pieza dental afectada (p = 0,078). No se encontró asociación estadísticamente significativa con el resto de las variables (Tabla 6).

**Tabla 4:** Distribución de frecuencias absolutas y relativas para las categorías distancia de la lesión apical a la cortical del seno maxilar con las variables categorizadas antropométricas y dentales

Variable	Categoría	Categoría de distancia de la lesión apical al seno maxilar (mm)						χ2	p
		Lesión yuxtapuesta		0 a 2		2 o más					
		n	%	n	%	n	%				
Sexo	Femenino	24	30,0	27	33,8	29	36,3	0,92	0,646
	Masculino	9	25,7	10	28,6	16	45,7		
Grupo etario (años)	20 a 39	10	22,2	14	31,1	21	46,7	3,19	0,535
	40 a 59	15	37,5	12	30,0	13	32,5		
	60 o más	8	26,7	11	36,7	11	36,7		
Seno maxilar	Derecho	20	26,0	23	29,9	34	44,2	2,49	0,309
	Izquierdo	13	34,2	14	36,8	11	28,9		
Pieza dental	SPM	6	27,3	7	31,8	9	40,9	2,94	0,580
	PM	25	31,6	23	29,1	31	39,2		
	SM	2	14,3	7	50,0	5	35,7		
Tratamiento endodóntico	Sí	12	26,7	14	31,1	19	42,2	0,31	0,887
	No	21	30,0	23	32,9	26	37,1		
Tamaño de la lesión apical (mm)	1 a 2	17	48,6	17	48,6	1	2,9	40,11	<0,001*
	2 a 4	15	29,4	15	29,4	21	41,2		
	4 a 8	1	3,4	5	17,2	23	79,3		

* significativo

**Tabla 5:** Distribución de frecuencias absolutas y relativas para el tipo de alteración del seno maxilar con las variables categorizadas antropométricas y dentales

Variable	Variable	Tipo de alteración del seno maxilar										χ2	P
		Sin alteración		Engrosamiento de la mucosa		Poliposis		Quiste mucoso de retención		Opacificación no específica					
		n	%	n	%	n	%	n	%	n	%				
Sexo	Femenino	10	12,5	43	53,8	13	16,3	10	12,5	4	5,0	3,41	0,505
	Masculino	3	8,6	24	68,6	4	11,4	4	11,4	0	0,0		
Grupo etario (años)	20 a 39	5	11,1	30	66,7	5	11,1	4	8,9	1	2,2	5,08	0,773
	40 a 59	6	15,0	22	55,0	6	15,0	5	12,5	1	2,5		
	60 o más	2	6,7	15	50,0	6	20,0	5	16,7	2	6,7		
Seno maxilar	Derecho	7	9,1	43	55,8	12	15,6	11	14,3	4	5,2	4,17	0,395
	Izquierdo	6	15,8	24	63,2	5	13,2	3	7,9	0	0,0		
Pieza dental	SPM	3	13,6	11	50,0	3	13,6	4	18,2	1	4,5	3,63	0,909
	PM	9	11,4	48	60,8	12	15,2	7	8,9	3	3,8		
	SM	1	7,1	8	57,1	2	14,3	3	21,4	0	0,0		
Tratamiento endodóntico	Sí	6	13,3	24	53,3	9	20,0	5	11,1	1	2,2	2,34	0,696
	No	7	10,0	43	61,4	8	11,4	9	12,9	3	4,3		
Tamaño de la lesión apical (mm)	1 a 2	3	8,6	29	82,9	3	8,6	0	0,0	0	0,0	59,79	<0,001*
	2 a 4	10	19,6	28	54,9	11	21,6	0	0,0	2	3,9		
	4 a 8	0	0,0	10	34,5	3	10,3	14	48,3	2	6,9		

* significativo

**Tabla 6:** Distribución de frecuencias absolutas y relativas para el estatus de la cortical inferior del seno maxilar con las variables categorizadas antropométricas y dentales

Variable	Variable	Estatus de la cortical inferior del seno maxilar										χ2	P
		Sin alteración		Adelgazada		Borrada		Desplazada		Discontinua					
		n	%	n	%	n	%	n	%	n	%				
Sexo	Femenino	13	16,3	34	42,5	15	18,8	6	7,5	12	15,0	12,57	0,012*
	Masculino	8	22,9	11	31,4	15	42,9	0	0,0	1	2,9		
Grupo etario (años)	20 a 39	11	24,4	13	28,9	12	26,7	2	4,4	7	15,6	6,57	0,600
	40 a 59	7	17,5	19	47,5	10	25,0	2	5,0	2	5,0		
	60 o más	3	10,0	13	43,3	8	26,7	2	6,7	4	13,3		
Seno maxilar	Derecho	12	15,6	31	40,3	21	27,3	4	5,2	9	11,7	1,15	0,910
	Izquierdo	9	23,7	14	36,8	9	23,7	2	5,3	4	10,5		
Pieza dental	SPM	2	9,1	11	50,0	3	13,6	3	13,6	3	13,6	14,06	0,078
	PM	17	21,5	31	39,2	22	27,8	3	3,8	6	7,6		
	SM	2	14,3	3	21,4	5	35,7	0	0,0	4	28,6		
Tratamiento endodóntico	Sí	7	15,6	22	48,9	10	22,2	3	6,7	3	6,7	4,22	0,389
	No	14	20,0	23	32,9	20	28,6	3	4,3	10	14,3		
Tamaño de la lesión apical (mm)	1 a 2	9	25,7	16	45,7	6	17,1	0	0,0	4	11,4	30,83	<0,001*
	2 a 4	12	23,5	22	43,1	9	17,6	1	2,0	7	13,7		
	4 a 8	0	0,0	7	24,1	15	51,7	5	17,2	2	6,9		

*significativo

## DISCUSIÓN

Como consecuencia de factores rinológicos y odontogénicos, pueden producirse cambios en la membrana sinusal, las más comunes suelen ser el engrosamiento y la opacificación. Desde el punto de vista odontológico, la interrelación entre las raíces de los dientes y la posibilidad de afectación del seno maxilar representa un determinante clave. Específicamente, la presencia de lesiones periapicales y su cercanía con el seno incrementan la probabilidad de un engrosamiento de la membrana sinusal. Aquí cobran importancia los procedimientos de imagen como la tomografía computarizada de haz cónico, que han hecho posible un aumento en la identificación de la etiología dentaria de esta situación clínica ^13^.

En relación con lo anterior, el interés del presente estudio fue evaluar la asociación entre cambios observados en los senos maxilares y las lesiones apicales mediante tomografía computarizada de haz cónico. Los resultados muestran que las unidades dentarias analizadas presentaron lesiones apicales en promedio 1,30 ± 0,53 y el 39,13% tenía tratamiento endodóntico. Se observaron lesiones apicales que promediaron un tamaño de 3,32 ± 1,82 mm, y casi la mitad (44,35%) presentó entre 2 y 4 mm de tamaño. La principal alteración del seno maxilar que se observó fue el engrosamiento de la mucosa (58,26%). El espesor promedio del engrosamiento de la mucosa sinusal fue de 3,51 ± 1,78 mm, con un 72,17% de los casos con engrosamientos mayores a 2 mm. Hubo asociación estadísticamente significativa entre las categorías de la distancia de la lesión apical a la cortical del seno maxilar con el tamaño de la lesión (p < 0,001), situación que los clínicos deben considerar para actuar a tiempo al momento de ubicar una patología específica.

Distintas investigaciones han analizado la relación del engrosamiento de la mucosa sinusal con la presencia de lesiones periapicales a través de la TCHC ^12^^,^^18^^,^^22^^-^^24^, los cuales reportan resultados que pueden compararse con el presente estudio. Por ejemplo, en esta investigación se reportó un espesor de la mucosa sinusal que, en promedio, era mayor de 3 mm, valor superior al evidenciado por Sghaireen ^23^, quien informó que en el 50% de los casos con patología periapical hubo un engrosamiento de la membrana asociado, es decir, se encontró que los dientes con lesión periapical tenían un engrosamiento de la membrana de Schneiderian significativamente mayor (p < 0,001), en comparación con los dientes adyacentes sanos. El engrosamiento medio de la membrana adyacente a las lesiones periapicales (2,45 ± 2,58) y variaba de 0 a 8 mm, mientras que el espesor iba de 0 a 5 mm del lado control donde no había lesión periapical asociada. 

Por otro lado, Aksoy y Orhan ^12^, reportaron un engrosamiento de la mucosa de más de 2 mm (grado 2 y grado 3) en uno o ambos senos maxilares en casi el 60% de sus pacientes. Evidenciaron una prevalencia de engrosamiento mucoso (> 2 mm) para senos maxilares con lesiones periapicales fue del 42,1% y sin lesiones periapicales, del 53,6% (p < 0,05). Dicha prevalencia aumentó en pacientes con pérdida ósea alveolar periodontal (p < 0,05). A diferencia del presente estudio, en su caso sí hubo una correlación significativa entre el engrosamiento de la mucosa con la edad, el sexo y la falta de dientes (p < 0,05).

Cabe mencionar, que Zhang *et al*. ^25^ reportaron un rango de engrosamiento mucoso de 8,25 ± 4,36 mm (2-16,6 mm). Entre los factores que afectan el grado este engrosamiento de la mucosa sinusal se encuentran la cantidad de pérdida de hueso alveolar (CPHA) y la altura mínima del hueso alveolar residual (AMHAR). La CPHA leve tuvo menos probabilidad que la CPHA grave y moderada de mostrar engrosamiento mucoso grave. Además, cuanto menor sea AMHAR, más grave será el engrosamiento.

Una publicación que se refiere a los factores de riesgo del engrosamiento de la mucosa de los senos maxilares es la de Huang *et al.*^24^, que reportaron una prevalencia total de engrosamiento de la membrana del seno maxilar del 36,6%. El engrosamiento de la membrana sinusal se asoció significativamente con pérdida ósea periodontal (p < 0,001) y lesiones periapicales (p < 0,001), respectivamente. El riesgo se calculó por medio de un modelo de regresión logística multivariable y se encontró que los hombres tenían un riesgo significativamente mayor de engrosamiento de la membrana sinusal que las mujeres (OR: 2,08, IC del 95% = 1,21-3,56). Además, los pacientes del grupo de edad ≥60 años mostraron un riesgo 4,35 veces mayor de engrosamiento de la membrana sinusal en comparación con los pacientes del grupo de edad ≤35 años.

En definitiva, según los resultados de la presente investigación puede afirmarse que hay un mayor riesgo de patología sinusal en pacientes con lesiones periapicales adyacentes al seno maxilar. De hecho, este aumenta a medida que aumenta el tamaño de la lesión, así como la cercanía con el seno. La tomografía computarizada de haz cónico representa un estudio de alta sensibilidad y especificidad para el diagnóstico de las lesiones del engrosamiento de la mucosa sinusal, y puede ayudar a corroborar si el origen es odontogénico o no. 

Este estudio no estuvo exento de limitaciones, ya que solo se contó con las imágenes evaluadas y no con los datos de una historia clínica que permitiera un análisis más global de la situación de cada uno de los casos.

## CONCLUSIONES

Existe asociación entre los cambios observados en los senos maxilares y las lesiones apicales, considerando que mientras más grande y cerca esta la lesión apical al seno maxilar, mayor es el engrosamiento de la mucosa sinusal.

## Fuente de financiamiento

Autofinanciado
